# Use of Transient Transfection for cGMP Manufacturing of eOD-GT8 60mer, a Self-Assembling Nanoparticle Germline-Targeting HIV-1 Vaccine Candidate

**DOI:** 10.3390/pharmaceutics16060742

**Published:** 2024-05-30

**Authors:** Vaneet K. Sharma, Sergey Menis, Evan T. Brower, Eddy Sayeed, Jim Ackland, Angela Lombardo, Christopher A. Cottrell, Jonathan L. Torres, Thomas Hassell, Andrew B. Ward, Vadim Tsvetnitsky, William R. Schief

**Affiliations:** 1 IAVI, New York, NY 10004, USA; vaneet.sharma@servier.com (V.K.S.); esayeed@iavi.org (E.S.); alombardo@iavi.org (A.L.);; 2 Servier Pharmaceuticals, Boston, MA 02210, USA; 3 IAVI Neutralizing Antibody Center, The Scripps Research Institute, La Jolla, CA 92037, USA; 4 Center for HIV/AIDS Vaccine Development, The Scripps Research Institute, La Jolla, CA 92037, USA; 5 Department of Immunology and Microbiology, The Scripps Research Institute, La Jolla, CA 92037, USA; 6 Paragon BioServices, Catalent Biologics, Baltimore, MD 21201, USA; 7 Global BioSolutions, P.O. Box 253, Vermont, VIC 3133, Australia; 8 Department of Integrative Structural and Computational Biology, The Scripps Research Institute, La Jolla, CA 92037, USA; 9 OncoC4, Inc., Rockville, MD 20850, USA; 10 The Ragon Institute of Massachusetts General Hospital, Massachusetts Institute of Technology and Harvard University, Cambridge, MA 02139, USA; 11 Moderna, Inc., Cambridge, MA 02139, USA

**Keywords:** transient transfection, cGMP, self-assembling nanoparticle vaccine, HIV vaccine

## Abstract

We describe the current Good Manufacturing Practice (cGMP) production and subsequent characterization of eOD-GT8 60mer, a glycosylated self-assembling nanoparticle HIV-1 vaccine candidate and germline targeting priming immunogen. Production was carried out via transient expression in the human embryonic kidney 293 (HEK293) cell line followed by a combination of purification techniques. A large-scale cGMP (200 L) production run yielded 354 mg of the purified eOD-GT8 60mer drug product material, which was formulated at 1 mg/mL in 10% sucrose in phosphate-buffered saline (PBS) at pH 7.2. The clinical trial material was comprehensively characterized for purity, antigenicity, glycan composition, amino acid sequence, and aggregation and by several safety-related tests during cGMP lot release. A comparison of the purified products produced at the 1 L scale and 200 L cGMP scale demonstrated the consistency and robustness of the transient transfection upstream process and the downstream purification strategies. The cGMP clinical trial material was tested in a Phase 1 clinical trial (NCT03547245), is currently being stored at −80 °C, and is on a stability testing program as per regulatory guidelines. The methods described here illustrate the utility of transient transfection for cGMP production of complex products such as glycosylated self-assembling nanoparticles.

## 1. Introduction

The goal of ending HIV/AIDS as a public health threat by 2030 envisaged for the world can only be realized by ensuring expanded and simplified HIV treatment and improved and effective use of interventions to prevent new infections [1]. Despite the steadily falling global human immunodeficiency virus (HIV) incidence and a reduction in the number of new infections to 1.7 million per year, there is a widespread consensus that to eradicate HIV, a vaccine is ultimately required [2].

Induction of broadly neutralizing antibodies (bnAbs), defined as antibodies capable of neutralizing diverse HIV isolates, is currently viewed as the central goal for HIV vaccine development. Results from antibody-mediated prevention (AMP) trials provided evidence that passive immunization with a bnAb can protect against HIV infection [3,4], consistent with similar studies in non-human primates [5]. Germline-targeting vaccine design is one of several approaches currently under evaluation for generating bnAb responses to the highly variable HIV envelope (Env) protein [6,7,8,9]. The germline targeting strategy is based on the design of priming immunogens that induce responses from rare bnAb precursor B cells. Sequential boosting with a series of immunogens then aims to guide antibody maturation to produce bnAbs [9,10,11,12].

VRC01-class bnAbs bind the CD4-binding site of HIV gp120 and include some of the most potent and highest breadth bnAbs known, making them important leads to guide vaccine design. At least three germline targeting immunogens designed to prime VRC01-class responses have been manufactured as proteins for clinical testing: eOD-GT8 60mer [13,14,15,16,17,18,19,20,21], 426c core nanoparticles [22,23], and BG505.SOSIP v4.1-GT1.1 [24,25].

While the prevention of HIV-1 infection with recombinantly produced bnAbs is conceivable, most experts in the field agree that the constantly evolving nature of the virus would invariably enable escape variants to appear quickly to defeat prevention efforts. To address this concern, more than one bnAb could be required for effective prevention. However, manufacturing of antibodies is still an expensive process and with current approaches, such treatments with multiple antibodies are bound to be unaffordable to many HIV patients [26]. Vaccination remains the best option for protecting most high-risk individuals from HIV infection.

Here we describe, for the first time, the cGMP manufacture of the HIV vaccine candidate eOD-GT8 60mer, a glycosylated self-assembling nanoparticle. In this molecule, the engineered outer domain of HIV gp120, germline targeting version 8 (eOD-GT8), is fused to the C-terminus of a modified version of the enzyme lumazine synthase from the bacteria *Aquifex aeolicus* through a flexible glycine-serine linker. The fusion protein sequence contains 341 amino acids and has a molecular weight of 36618.1 Da. There are eight paired cysteines and ten N-linked glycosylation sites in the sequence. Upon secretion from mammalian HEK293 cells, the recombinant protein assembles into icosahedral nanoparticle structures (~30 nm diameter, ~3000 kDa molecular weight) composed of 60 identical subunits. The assembly process is driven by the inherent propensity for self-association of the lumazine synthase.

Although transient transfection is widely used in manufacturing of viral vectors and protein production to make non-clinical materials [27,28], this approach is not used in the industry for generating protein products in cell culture under cGMP conditions for clinical trials, where fed-batch processes are routinely used as standard. To produce eOD-GT8 60mer, key components of the upstream processing include the growth of suspension-adapted HEK293H cells, transient transfection components and conditions, a feed strategy, and media and culture conditions. In the downstream processing the clarified harvest material was subjected to Benzonase treatment, viral inactivation (solvent/detergent treatment), purification via primary column chromatography on anion-exchange resin followed by polishing chromatography using ceramic hydroxyapatite resin, and nanofiltration-based virus reduction. Analytical characterization for the cGMP-produced eOD-GT8 60mer clinical trial material was based on established US Food and Drug Administration (FDA) requirements and International Conference on Harmonization (ICH) guidelines to determine its safety, identity, concentration, purity, in vitro potency and stability [29,30,31]. Additional characterization included amino acid analysis (LC-MS/MS peptide mapping), N-linked glycan occupancy analysis (HILIC-FLD-MS), monosaccharide compositional analysis using RP-HPLC, particle size (DLS), and aggregation analysis using analytical ultracentrifugation (AUC). Additionally, a nonclinical GLP repeat-dose toxicity study was conducted in rabbits prior to a Phase 1 clinical trial in healthy volunteers (NCT03547245).

## 2. Materials and Methods

The eOD-GT8 60mer clinical trial material was manufactured at Paragon BioServices, Inc (Baltimore, MD, USA, now part of Catalent Biologics under the name Paragon Gene Therapy. A cGMP facility was used to manufacture the clinical trial material using multiple unit operations, sterile techniques, and standard operation procedures. A working cell bank (WCB) of the suspension-adapted HEK293 cells, referred to as VRC293, was generated at SAFC (Carlsbad, CA, USA) from a master cell bank (MCB) generously provided by the National Institute of Health (NIH)/National Institute of Allergy and Infectious Diseases (NIAID)/Vaccine Research Center (VRC). The cGMP-qualified VRC293 cell line was generated from the HEK293H cell line at the VRC [32,33]. The VRC293 WCB was tested for the absence of adventitious agents at release and for sterility and lack of mycoplasma contamination before cGMP process use. A vial of WCB was thawed and expanded for the manufacturing process. The plasmid DNA (pDNA)-encoding eOD-GT8 60mer polypeptide chain was manufactured at Aldevron (Fargo, ND, USA). Kifunensine, a chemically synthesized alkaloid, was manufactured under cGMP conditions by GlycoSyn (Gracefield, New Zealand). Benzonase^®^ endonuclease enzyme (high-purity grade, 250 U/μL) was purchased from Millipore (Burlington, MA, USA). Polyethylenimide PEIpro -HQ^®^ transfection reagent, a chemically synthesized polymer, was manufactured under cGMP conditions by PolyPlus-Transfection SA (Illkirch, France). Expi293 medium was purchased from Thermo Fisher Scientific (Waltham, MA, USA).

### 2.1. Transient Transfection and Upstream Process Development

The upstream process aimed to achieve a high cell number, product titer and quality in the harvest [34,35]. VRC293 cells were expanded to grow in suspension in serum-free Expi293 media supplemented with 4 mM L-glutamine through a total of 8 passages via 1.0 L and 3.0 L shake flasks and a 25 L Wave bioreactor (GE Healthcare, Chicago, IL, USA) into a Biostat Cultibag STR 200 Plus (Sartorius Stedim Biotech, Aubagne, France) single-use production bioreactor. The cells, in an approximately 180 L working volume, were transiently transfected with the PEIpro-HQ^®^/pDNA complex at a cell density of ca. 10^6^ cells/mL (at 97.9% viability). To achieve a high transfection efficiency, a solution containing a 2:1 mass ratio of PEIPro-HQ^®^ to pDNA was incubated for 12–15 min at ambient temperature and then added to VRC293 cells in OptiPRO FreeStyle serum-free medium (Gibco; Thermo Fisher Scientific (Waltham, MA, USA) at 37 °C. To fulfill regulatory requirements for using high-quality GMP source pDNA (Aldevron) quality control analytical testing was performed to ensure pDNA was sterile, of high purity, and at the desired concentration. Additionally, tests were conducted to ensure that supercoiled pDNA was essentially free from genomic DNA [analysis of residual host cell *Escherichia coli* chromosomal DNA was estimated by quantitative real-time polymerase chain reaction (qPCR) and passed specifications], host cells proteins [analysis by Micro BCA (bicinchoninic acid chromogenic) assay passed specifications <1% protein content], residual RNA (analysis by Agarose Gel Electrophoresis with SYBR^®^ gold staining passed specifications), and endotoxins (analysis by LAL kinetic turbidimetric assay passed specifications <2.5 EU/mg).

Kifunensine solution was added to the bioreactor after transfection to a final concentration of 14 µM to inhibit type-1 α-mannosidases in the endoplasmic reticulum (and likely also in the Golgi) and thereby maintain high-mannose glycoprotein levels during the transient transfection stage [17,36,37]. Culture pH, temperature, dissolved oxygen, and agitation in the bioreactor were maintained post-transfection at pH 7.2 ± 0.1, 37 ± 2 °C, 39 ± 3% dissolved oxygen, and 75–90 rpm, respectively. During the run, cell density, cell viability, pH, and glucose and glutamine levels were monitored daily using a Vi-CELL™ XR Cell Viability Analyzer (Beckman Coulter, Inc., Fullerton, CA, USA) and a BioFlex instrument (Nova Biomedical, Waltham, MA, USA). Glucose and glutamine were supplemented after transfection as needed to avoid depletion. D-glucose (40%) was added on days 3 and 5 and L-glutamine (200 mM) was added on day 3 to maintain their respective concentrations (glucose ≥ 2 g/L and L-glutamine ≥ 2 mM). The cumulative amounts of glucose and glutamine added post transfection to the bioreactor were 4 kg and 3 kg, respectively. In the bioreactor, culture conditions such as the impeller speed, pH, and temperature were also monitored daily. Seven days post transfection, the cells were separated from the conditioned supernatant by Unifuge (Pneumatic Scale Angelus, Stow, OH, USA), a fully automated centrifuge system that uses a single-use insert inside a centrifuge bowl. Opticap XL capsule filters (EMD Millipore, Burlington, MA, USA) were used for further cell separation and harvest clarification. The resulting clarified harvest was filtered through a 0.22 µm Millipore filter, and the product titer was determined to be 42.5 mg/L by a product-specific enzyme-linked immunosorbent assay (ELISA).

### 2.2. Downstream Process Development

**Figure 1:**
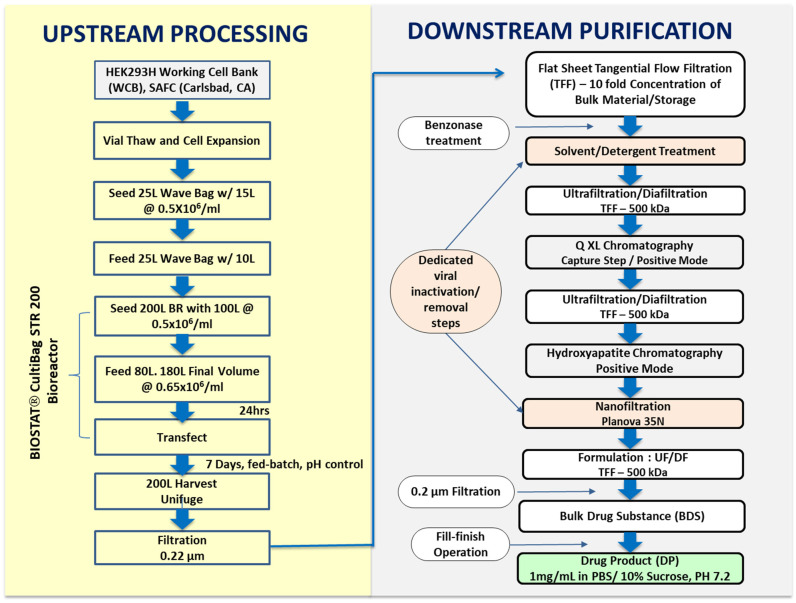
Process flow diagram for eOD-GT8 60mer manufacturing using transiently transfected HEK293H cells.

The eOD-GT8 60mer manufacturing process, depicted in Figure 1, was focused on removing the process-derived and product-derived impurities and reducing bioburden and endotoxin, while providing an acceptable product yield [38,39]. The clarified harvest was concentrated approximately ten-fold by flat sheet tangential flow filtration (TFF) filters with a molecular cut-off of 500 kDa (EMD Millipore). The flat sheet filters had a combined surface area of 4 m^2^. The concentrated harvest was treated with endonuclease to digest any remaining host cell and plasmid DNA to facilitate meeting the regulatory threshold for DNA contamination. Sucrose was added to 10% (*w*/*v*) final concentration before the storage of the cell culture harvest at −80 °C.

Although HEK293 cells are not known to harbor known endogenous retroviruses and adventitious agents, assessed by Cytopathogenic Effect (CPE), Hemadsorption test (HAD) and Hemagglutination (HA) test using MRC-5, Vero and HEK292 cells, were not detected in VRC92 MCB and WCB (Charles River, Malvern, PA, USA), a solvent–detergent treatment step was incorporated in the manufacturing process out of an abundance of caution for patients’ safety. The step was designed to inactivate enveloped viruses [40]. Tri-n-butyl phosphate and Tween 80 were added to the Benzonase-treated cell harvest to 0.3% and 1% final concentrations, respectively, and the solution was allowed to incubate for 16–20 h at 2–8 °C.

Q Sepharose XL Anion Exchange Chromatography: After endonuclease and solvent–detergent treatment, the clarified harvest was diafiltered into loading buffer (20 mM HEPES, 50 mM NaCl, pH 7.4) and loaded onto a 30 cm (i.d.) BPG™ 300 column (Amersham Biosciences Piscataway, NJ, USA) packed with 1350 mL of Q-Sepharose XL resin (10 cm bed height) to perform anion exchange chromatography in bind-and-elute mode. Following column washing, eOD-GT8 60mer captured by the resin was eluted isocratically using five column volumes of 20 mM HEPES and 250 mM NaCl buffer at pH 7.4 (flow rate 30 cm/h). In addition to providing enrichment of eOD-GT8 60mer, this chromatography step served to reduce the levels of contaminating DNA in the eluate. Even though the VRC293 cell line was derived from human tissue, residual host-cell protein and host-cell DNA in the eOD-GT8 60mer product needed to be reduced to acceptable threshold values, typically below 100 µg/dose and 10 ng/dose (with DNA fragments not exceeding 200 base pairs), respectively, as per CBER guidelines, 2007.

Ceramic Hydroxyapatite (CHT) Column Chromatography: Ceramic hydroxyapatite (CHTTM, type 1, Bio-Rad, Hercules, CA), a crystalline mineral [(Ca_5_(PO_4_)_3_OH)_2_] with multimodal functionalities, was used in the bind-and-elute mode as a polishing step to remove aggregates and other product-related impurities. A CHT type 1 resin (80 μm particle size) packed in a BPG™ 300 column (Amersham Biosciences Piscataway, NJ) was used to perform further eOD-GT8 60mer purification. Before the chromatography step, the packed column (6.3 L in volume) was equilibrated with 5 mM sodium phosphate, pH 7.2 equilibration buffer (≥4 CV). Prior to loading the Q Sepharose eluate onto the CHT column, the material was diafiltered into 5 mM sodium phosphate, pH 7.2 buffer. Under these conditions, the amino groups of the eOD-GT8 60mer glycoprotein would be expected to bind to the phosphate ions of the mineral via a classical cation exchange mechanism, and carboxylic groups would be expected to bind to the Ca^2+^ ions of the resin by a combination of calcium metal affinity and anion exchange. Bound eOD-GT8 60mer glycoprotein was eluted from the CHT resin with a linear gradient from 0 to 100% of 500 mM sodium phosphate, pH 7 buffer, over 10 column volumes at 9.4 L/hr. Fractions enriched with the target protein were pooled based on information obtained during the process development at small scale and an earlier engineering run performed at the full 200 L production scale.

Viral Nanofiltration: The post-CHT pool was nanofiltered using single-use Planova 35N (35 nm nominal pore size, surface area 0.12 m^2^) membranes (Asahi Kasei Corporation, Tokyo, Japan) as a virus-removing polishing step, which complemented the solvent/detergent viral inactivation operation performed earlier in the downstream purification. A formal viral clearance study demonstrated that for model viruses, Planova 35N filtration provided > 3.83 log clearance for the xenotropic murine leukemia virus (XMuLV, 70–100 nm retrovirus) and 6.39 log clearance for the bovine viral diarrhea virus (BVDV, 40–70 nm pestivirus). After viral nanofiltration, the filtered CHT pool was TFF-formulated into a bulk drug substance (DS) by concentrating the eOD-GT8 60mer to 1.0 mg/mL and buffer-exchanging to 10 mM Na_2_HPO_4_, 2 mM NaH_2_PO_4_, 137 mM NaCl, 2.7 mM KCl, 10% sucrose at pH 7.4. A total of 354 mg of the eOD-GT8 60mer DS was purified from the starting 200 L cGMP batch (4.3% process yield).

The formulated purified drug substance was filter-sterilized through a 0.22 μm filter (PVDF, Millipore, Burlington, MA, USA) into a sterile single-use bag. The clinical trial material was filled at a 0.4 mL volume in 2 mL Type 1 glass vials with stoppers (13 mm stopper, Rubber with Flurotec, Afton Scientific) and sealed with sterile seals from Afton Scientific Corporation (Charlottesville, VA, USA). Every sealed vial was subjected to visual inspection; vials were labeled and packaged into allocated fiberboard freezer boxes and kept at the intended storage temperature (−80 °C).

### 2.3. Analytical Characterization

**Figure 2:**
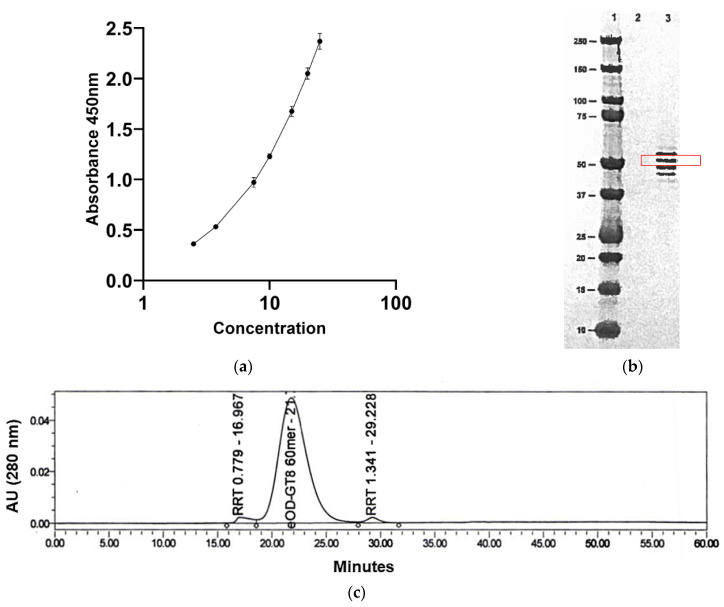

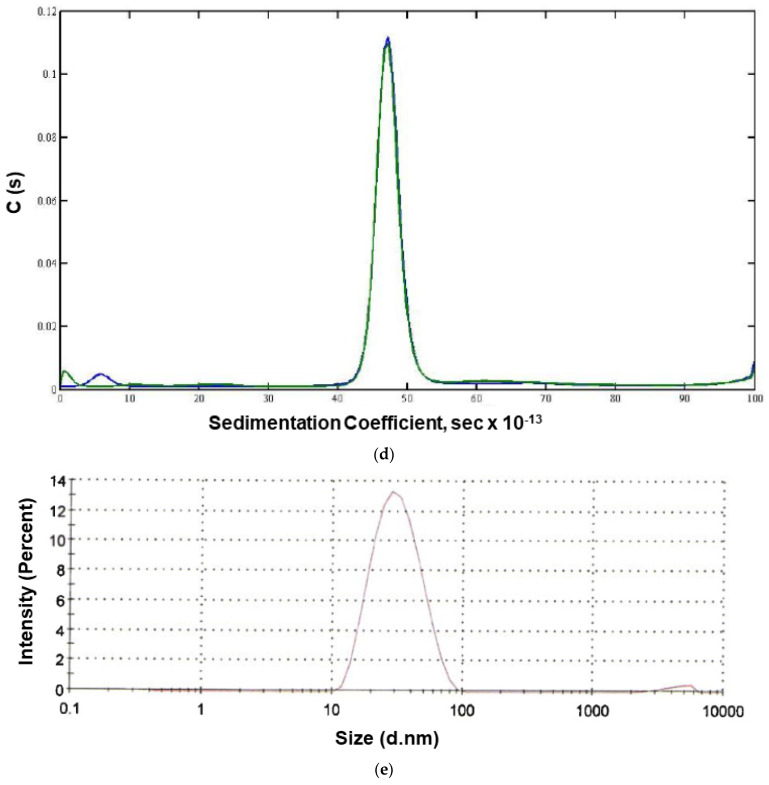
Analytical characterization of the eOD-GT8 60mer. (**a**) Sandwich ELISA standard curve for the in vitro potency analysis. (**b**) N-terminal sequencing by Edman degradation. The band enclosed in the red rectangle was excised. (**c**) SE-HPLC analysis to monitor product purity. (**d**) AUC-SV analysis to determine peak distribution. (**e**) Dynamic light scattering (DLS) to determine average particle size via size distribution by intensity.

A sandwich ELISA was developed to characterize the potency of the eOD-GT8 60mer glycoprotein (Figure 2a). This assay measured the binding of the germline (GL) VRC01 antibodies to the eOD-GT8 60mer nanoparticle. ELISA was performed using 96-well Maxisorp ELISA plates (Thermo Fisher Scientific), coated overnight with 100 µL/well of 4 μg/mL GL-VRCO1 Fab (produced in-house at The Scripps Research Institute, San Diego, CA, USA). The plates were incubated at 2–8 °C for 18 ± 2 h, followed by washing each plate three times with the wash buffer (PBS, 0.05% Tween 20) and subsequently blocking at 25 °C for 45 ± 2 min while rotating at 180 RPM with 200 µL of BSA blocking buffer (PBS, 0.05% Tween 20, 2% BSA). After incubating with serially diluted samples containing eOD-GT8 60mer, 100 µL of 100 ng/mL of GL-VRC01 antibody solution was added to each well and incubated at 25 °C for 45 ± 2 min at 180 RPM. This process was followed by adding 100 µL of diluted secondary HRP-conjugated goat anti-human IgG Fcγ antibody to each well and incubating at 25 °C for 45 ± 2 min at 180 RPM. After three washes with wash buffer, 100 µL of TMB substrate (Sigma Aldrich, St. Louis, MO, USA) was added to develop the chromogenic signal (10 min in the dark, 25 °C). The reaction was stopped with 100 µL of 2N sulfuric acid, and the absorbance at 450 nm was measured using a Spectramax microplate reader. The results were analyzed using Softmax Pro software (Molecular Devices LLC, Sunnyvale, CA, USA, https://www.moleculardevices.com/, accessed on 7 May 2024). The in vitro relative potency was expressed as a percentage and defined as the ratio of protein concentration (determined by an ELISA) to protein concentration (determined by measuring the absorbance at 280 nm). For all tested eOD-GT8 60mer samples, potency was determined to be between 89 and 100% (Table 1).

The eOD-GT8 60mer amino acid sequence was confirmed using Agilent 1290 Infinity II HPLC coupled to an Agilent 6550 QTOF Mass Spectrometer. A Waters XBridge CSH C18 column (130 Å, 1.7 μm, 2.1 × 150 mm) was used to perform chromatographic separation. The mobile phases used were 0.1% formic acid (mobile phase A) and 0.1% formic acid in 100% acetonitrile (mobile phase B). Bottom-up proteomics techniques (enzymatic digestion followed by MS analysis of proteolytic peptide mixtures) were used to perform peptide mapping. The eOD-GT8 60mer glycoprotein was reduced and carboxymethylated followed by lysine-C (Lys-C)/trypsin sequential digestion and treatment with Glutamyl endopeptidase (Glu-C). The MS data were acquired in positive ionization mode over the range between 225 and 2500 *m*/*z* and MS/MS fragmentation data were acquired over the range between 100 *m*/*z* and 3000 *m*/*z*. The total ion chromatograms were analyzed using Agilent MassHunter software, and sequence matching was carried out using Agilent BioConfirm software (https://www.agilent.com/en/product/software-informatics/mass-spectrometry-software/data-analysis/bioconfirm-software, accessed on 7 May 2024). For all tested eOD-GT8 60mer samples, over 95% f the amino acid sequence was positively mapped, including the N- and C-termini sequences, and similar PTMs (deamidation, oxidation, pyroglutamylation, and N-linked glycosylation) were identified (Table 1).

N-terminal amino acid sequencing for the eOD-GT8 60mer was performed using the Procise Protein Sequencing System (Applied Biosystems, Waltham, CA, USA). The protein band corresponding to the eOD-GT8 60mer (~50 kDa) was excised from SDS-PAGE gel (Figure 2b), transferred to a clean Eppendorf tube and subjected to 10 cycles of Edman degradation as described by Tempst and colleagues [41]. The N-terminal amino acid sequence was determined to be ETGMQIYEGK for the non-clinical (200 L) and cGMP (200 L) batch (Table 1). The ETG residue on the N-terminal side for the non-clinical and cGMP batch remained from the secretion leader sequence after cleavage of the leader sequence. Additional quality attributes critical to the eOD-GT8 60mer structure were evaluated and are listed in Table 1. Size exclusion–high-pressure liquid chromatography (SEC-HPLC) was performed to monitor product purity. SEC-HPLC was carried out using an Alliance e2695 HPLC system (Waters Co., Milford, MA, USA) equipped with a Superose 6 Increase 10/300 GL column (GE Healthcare Life Sciences) and a 2998 photodiode array (PDA) detector. A total of 100 µg of the sample was directly injected onto the column at 25 °C and a mobile phase consisting of 10 mM HEPES and 150 mM NaCl, pH 7.4, was used. The flow rate was 0.5 mL/min, and the main peaks of 60mer and impurities were detected under native conditions with UV detection at 280 nm. Data was acquired and processed by the Empower™3 (Waters) software (https://www.waters.com/nextgen/us/en/products/informatics-and-software/chromatography-software/empower-software-solutions.html, accessed on 7 May 2024).

The size distribution analysis of eOD-GT8 60mer samples was carried out by sedimentation velocity analytical ultracentrifugation (AUC-SV) on a Beckman Optima XL-A Ultracentrifuge equipped with a four-hole rotor [42]. A sample held within one of the two channels in a 12 mm double-sector Epon centerpiece was sedimented at a moderate speed (20,000 rpm). All the tested samples on SEC-HPLC and AUC showed no aggregates and only one main peak corresponding to the eOD-GT8 60mer (Figure 2c,d) without any evidence of particle disassembly into smaller pentameric or monomeric components. The average particle size of eOD-GT8 60mer was measured as 29 nm with a PDI of <0.3 (Figure 2e) via dynamic light scattering (DLS) with the Zetasizer NanoZS (Malvern, Southborough, MA, USA) [43].

Long-term stability studies for the cGMP batch of the eOD-GT8 60mer clinical trial material were designed according to the ICH Q5A guideline. The stability of the clinical trial material stored at −80 °C continues to be monitored over a 36-month period. Additionally, short-term stability studies were conducted for liquid formulations stored at 5° ± 3 °C (0, 24 h) and at 25 °C (0, 24 h). At each time point, testing was performed to evaluate the appearance, pH, protein concentration, purity (SE-HPLC and SDS-PAGE), and relative potency. All the data generated to date (up to 36 months) demonstrate that the material continues to meet release specifications and no adverse trends in the stability-indicating parameters have been detected.

## 3. Results

In the biopharmaceutical industry, comprehensive analytical characterization is required to support a product through development and cGMP manufacturing [29,30,31,44,45]. It is also a regulatory requirement to perform analytical testing on the cGMP-manufactured clinical trial material [46,47]. Unlike most proteins such as monoclonal antibodies (~150 kDa) or other candidate HIV-1 vaccine immunogens (~120–400 kDa), eOD-GT8 60mer megadalton nanoparticles (~3000 kDa, ~30 nm diameter) posed significant characterization challenges to many traditional analytical quality control (QC) methods [48]. QC procedures were performed on the manufactured eOD-GT8 60mer nanoparticle to confirm its identity, determine protein concentration and purity, establish in vitro potency and measure the nanoparticle size. Additionally, to help assure safety for clinical trial participants, the clinical trial lot was tested to quantify host cell residual impurities (host cell proteins and host cell DNA), measure bioburden and bacterial endotoxin, determine subvisible particulate matter, and confirm sterility.

Although a specification-based analytical control strategy was established to ensure the quality and safety, the complex nature and composition of eOD-GT8 60mer nanoparticles could lead to product variation influenced by upstream conditions or downstream purification processes. To address this possibility, high-resolution analytics were carried out to physiochemically characterize the eOD-GT8 60mer nanoparticles [29,42,43,44,49,50]. Overlapping information derived from the orthogonal analytical assays not only facilitated a better understanding of the structure and composition of the eOD-GT8 60mer clinical trial material, but also assured product safety.

As the transient-transfection-based process was developed at a 2.0 L scale, optimized at 10.0 L volume, and further scaled-up to the 200 L cGMP clinical batch, the product critical quality attributes (CQAs) were established and monitored during scale-up to demonstrate product consistency. A combination of biophysical, immunochemical, and physicochemical methods was used to perform comparability testing to assess antigenicity, three-dimensional structures, post-translational modifications, and protein aggregation. A systematic characterization performed throughout the project validated the reliable and reproducible scale-up, and batches of the eOD-GT8 60mer nanoparticles produced at different scales demonstrated near identical CQAs.

**Figure 3:**
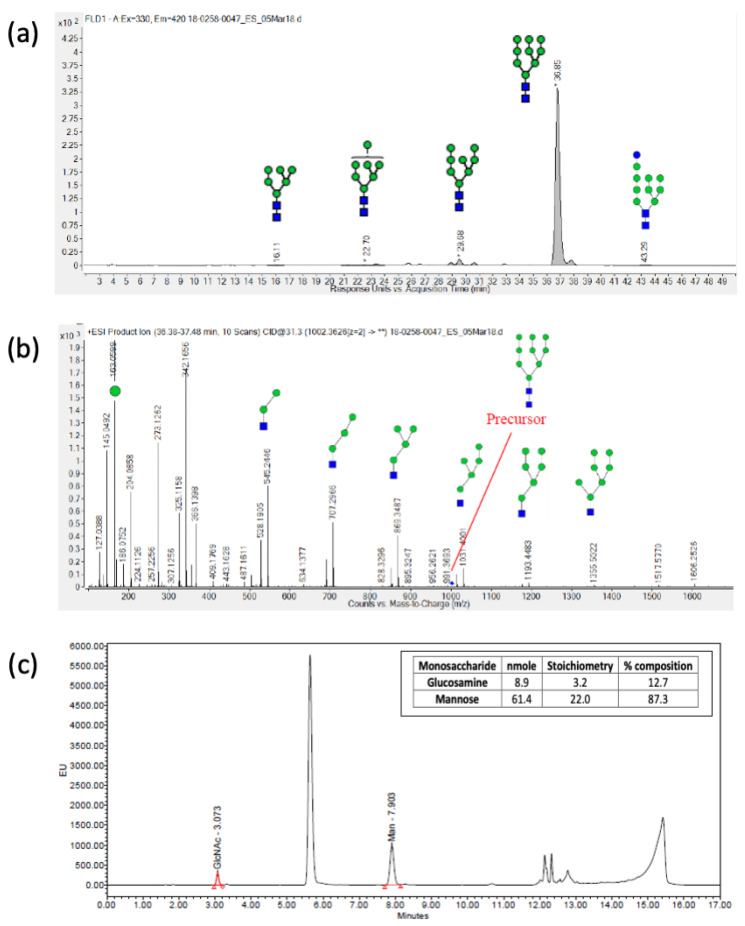
N-linked glycan analysis of the eOD-GT8-60mer sample: (**a**) Released N-glycans analysis using UPLC-HILIC-FLD. Man9 (95.5%) was determined to be the most dominant N-linked glycan. (**b**) MS/MS of Man9 using Q-TOF/MS. (**c**) Quantitative monosaccharide composition analysis using RP-HPLC.

**Figure 4:**
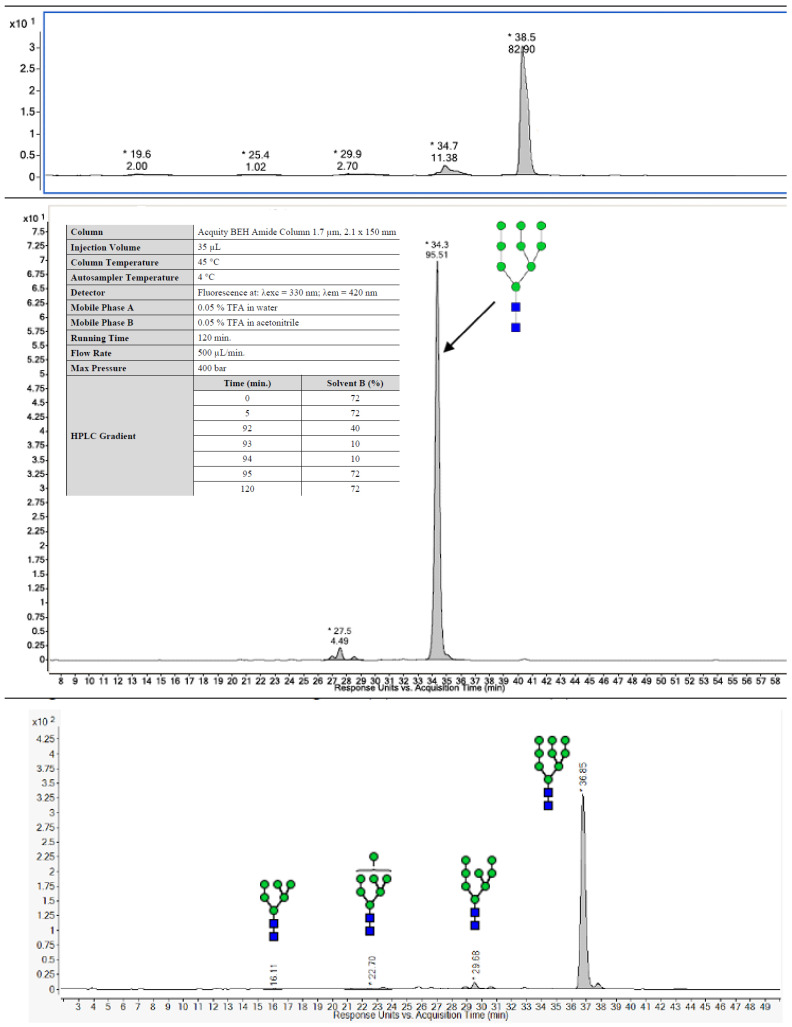
N-linked glycan comparison for the process (10 L), non-clinical batch (200 L) and clinical batch (200 L) scale runs. The difference in the retention time is due to different HPLC methods used. The run conditions were identical, detailed in the middle panel. The N-linked glycan structure was confirmed by MS/MS.

N-linked glycan profiling was performed at Charles River Laboratories using hydrophilic interaction liquid chromatography coupled to mass spectrometry (HILIC-FLD-MS/MS) to determine the type of N-linked glycans present on the eOD-GT8 60mer (Figure 3a). A two-step N-glycan release procedure was implemented to ensure the completeness of the deglycosylation reaction. Samples were first treated by non-denaturing release overnight with N-glycinate (Prozyme) at 37 °C and, in a second step, a denaturation buffer containing SDS was applied prior to another overnight N-glycanase digestion. The released glycans were 2-AB labeled (Sigma-Aldrich, St. Louis, MI, USA) and quantified using HILIC-FLD equipped with a fluorescence detector (excitation and emission wavelengths of 330 nm and 420 nm, respectively). The eluting N-linked glycans were further characterized using a high-resolution Agilent ESI-QTOF mass spectrometer, model 6550. Man9 [(*Man*)*9*(*GlcNAc*)*2*] was the most abundant N-linked glycan for all tested samples (Figure 4, Table 1). 

A high-resolution LC-MS/MS peptide-mapping-based method was used to determine the sequence coverage and identity of N-linked glycosylation sites. The sample was treated with PNGase-F to remove N-glycans and digested with the trypsin coupled with Lys-C followed by GluC. The occupancy of N-glycans at each potential site was determined from the MS1 and MS2 data extracted from MS raw files processed using the Agilent Masshunter software (https://www.agilent.com/en/promotions/masshunter-mass-spec, accessed on 7 May 2024) and BioConfirm tools. The MS2 analysis reduced misinterpretation of glycosylation site occupancy by MS1 data alone from potential deamidation of Asn and/or Gln residue. LC-MS/MS analysis assessed de-N-glycosylation producing Asn to Asp conversion and a corresponding mass shift of +0.98 Da to identify the potential N-glycan sites as occupied, partially occupied, or unoccupied using a similar method that is less quantitative in measuring occupancy than methods described by Paulson and colleagues [49,50,51]. Apart from some minor differences, all the tested samples (Table 1) exhibited a similar sequence coverage and N-glycan occupancy. Five N-glycan sites were fully occupied, three were partially occupied, and two were not occupied.

In addition to the N-linked glycan analysis, a monosaccharide analysis was also performed to confirm the composition of the sugars in the eOD-GT8 60mer glycoprotein [52]. The test sample was hydrolyzed using trifluoroacetic acid (TFA) and then derivatized using fluorescent 2-aminobenzoic acid (2-AA) prior to reverse-phase chromatographic analysis (RP-HPLC). The separated monosaccharide derivatives were detected by their fluorescence intensity using ultra-high-performance liquid chromatography (*UPLC*-*FLD*) linked with a fluorescence detector (excitation and emission wavelengths of 360 nm and 425 nm, respectively). In all tested samples, mannose and glucosamine were the only two sugars detected (Figure 3b, Table 1).

**Figure 5:**
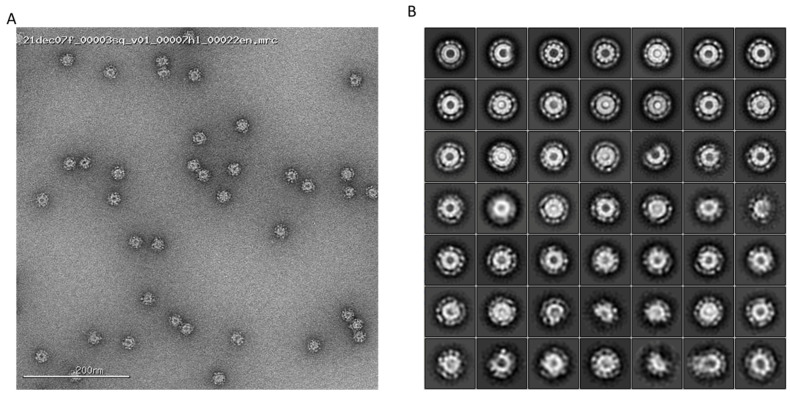
Negative stain transmission electron microscopy: (**A**) Representative micrograph of preclinical eOD-GT8 60mer nanoparticles. (**B**) Two-dimensional class averages of eOD-GT8 60mer nanoparticles.

For negative stain electron microscopy, eOD-GT8 60mer samples were diluted to 0.01 mg/mL in 1X TBS pH 7.4, deposited onto a glow discharged carbon-coated copper mesh grid, and stained for 90 sec with 2% uranyl formate. An FEI Tecnai Spirit T12 (120 keV at 56 kx magnification) paired with an FEI Eagle 4k × 4k CCD camera was used for data collection. Data collection automation was made possible by Leginon software (https://emg.nysbc.org/projects/leginon/wiki/Leginon_Homepage, accessed on 7 May 2024) and Appion (https://emg.nysbc.org/projects/appion/wiki/Appion_Home, accessed on 7 May 2024) was used to store the resulting micrographs. DoGpicker (https://sbgrid.org/software/titles/dogpicker, accessed on 7 May 2024) was used to pick particles from the micrographs and RELION 3.04 aligned the particles into 2D classes. The data demonstrate a homogeneous nanoparticle shape and distribution (Figure 5).

The multifaceted eOD-GT8 60mer nanoparticle characterization revealed similar product characteristics among different batches. The analytical testing results for batches produced at a different scale (i.e., 1.0 L research run, 20 L process run, 200 L engineering run, and 200 L cGMP batch) for the specified attributes are summarized in Table 1.

### Nonclinical Safety Assessment

The objectives of this study were to determine the potential toxicity and local tolerance of the eOD-GT8 60mer/AS01_B_ vaccine when given via intramuscular (IM) injection and of the eOD-GT8 60mer when given via intramuscular or subcutaneous (SC) injection. The vaccine products were given once every two weeks for four doses to New Zealand White rabbits. The following parameters and end points were evaluated in this study: clinical signs, dermal scores, body weights, body weight gains, food consumption (males only), ophthalmology, body temperature, clinical pathology parameters (hematology, coagulation, clinical chemistry, and c-reactive protein) and immunogenicity (antibody analysis).

Intramuscular injection of the OD-GT8 60mer/AS01B vaccine was well tolerated and was only associated with minor disturbances in clinical pathology, higher c reactive protein levels, and findings of mixed inflammatory cell infiltrates at the administration sites. Intramuscular or subcutaneous injection of eOD-GT8 60mer was well tolerated and was only associated with findings of mixed inflammatory cell infiltrate at the administration sites. After a three week treatment-free period, the findings were no longer recorded or were at a lower incidence and severity. There were no adverse findings.

## 4. Discussion

Transient transfection is a common laboratory technique to quickly produce small amounts of protein from mammalian cell cultures. While it is widely used now at larger scales to produce clinical supplies of viral vectors for gene therapy, production of clinically relevant proteins using transient transfection has not been reported. Here, we describe a reproducible and scalable transient-transfection-based process for GMP manufacturing of an HIV-1 vaccine priming candidate with complex molecular features (glycosylated, self-assembling nanoparticles), and we detail an analytical testing strategy for product characterization and release.

It is important, but challenging, to maintain a comparable process performance for each unit operation upon scale up. During transient transfection, plasmid DNA must be mixed with a transfection reagent and then added to the bioreactor. Fluid dynamics in different bioreactor volumes are a concern, as there is a narrow window of time in which mixing and subsequent addition to the cells should be achieved to maintain a consistent transfection efficiency and production yield. Here, we demonstrate that transient transfection of HEK293 cells with polyethylenimine (PEI) as a transfection reagent at 2, 10 and 200 L scales provided very similar product titers in the harvest and an indistinguishable quality of the final product after downstream purification. The process conditions employed for the engineering run conducted at a full 200 L scale were then faithfully reproduced for the cGMP run at the same scale, resulting in product batches with the same product characteristics.

In the upstream process, consistency of production was achieved by identifying conditions that minimized cell clumping and ensuring optimal feeding to maintain a high HEK293 cell viability. The downstream process allowed for efficient purification of the megadalton nanoparticle from process- and product-related impurities. The eOD-GT8 60mer bound efficiently to the anion exchange resin in the first chromatography step, allowing for significant enrichment in the eluate, and the subsequent hydroxyapatite column provided efficient separation from remaining impurities. The two-column process was supplemented with viral inactivation and removal steps. Additionally, we took advantage of the product large molecular weight and utilized a large-pore-size TFF membrane for buffer exchange and concentration steps, while achieving some additional polishing.

We utilized an extensive analytical toolbox and sophisticated techniques to better understand the product characteristics and to obtain baseline data for future manufacturing batches. In addition to confirming the identity, potency and safety of the clinical product, we demonstrated the consistent average size of the eOD-GT8 60mer nanoparticles and a lack of aggregation. For consistency with preclinical experiments, Kifunensine was used to ensure that the glycans would be high mannose. N-linked glycan profiling of the product confirmed the predominance on Man9 among the eluted sugars. N-linked glycan occupancy analyses showed that five of the ten N-linked glycosylation sites were partially or fully unoccupied. Preclinical material, produced by transient transfection in 293 cells and purified using lectin affinity followed by size exclusion chromatography, had seven N-glycan sites partially or fully unoccupied [19], but performed well in multiple mouse models [14,15,17,20,21,53,54]. We viewed the underoccupancy of glycans as unlikely to hamper performance in human clinical testing. Whether glycan occupancy would be improved by production from a stable cell line is not known. As quality control analytical testing alone does not ensure product safety, a non-clinical safety assessment of the eOD-GT8 60mer produced at a 200 L batch scale was performed to increase the assurance of safety. There were no findings considered to be adverse.

The pursuit of a safe and effective HIV vaccine is a significant public health priority. The work described here supported the clinical testing of a new immunogen, eOD-GT8 60mer, and a new vaccine concept, germline targeting [9,55]. A randomized, double-blind, placebo-controlled dosage-escalation Phase 1 study was conducted to assess the safety, tolerability, and immunogenicity of the eOD-GT8 60mer vaccine, adjuvanted with AS01_B_, in up to 48 healthy adult HIV-negative volunteers (ClinicalTrials.gov Identifier: NCT03547245). The adjuvanted protein was found to induce the targeted VRC01-class bnAb precursors to substantial frequencies in the blood of 97% of vaccine recipients [9], supporting not only the germline-targeting concept and the specific molecular design of eOD-GT8 60mer but also the cGMP process described here. Experimental medicine clinical trial studies to evaluate new HIV vaccine candidates are novel, and manufacturing these products in accordance with cGMP is challenging. Manufacturing of highly pure and stable eOD-GT8 60mer from a cGMP process based on transient transfection of HEK293 cells provides a potential template for cGMP manufacture of other immunogens. Relatively small amounts of the produced drug product were sufficient for lot release and stability testing, and, given the low clinical dose, provided enough material for potentially several Phase 1 studies. The reliability and robustness built through comprehensive analytical characterization have established procedures that are adaptable to successful cGMP campaigns for future immunogen-based HIV vaccine projects.

It is worth noting that while the method we describe may be broadly applicable to manufacturing other recombinant proteins for initial clinical testing, it is more likely to be used in niche situations due to its limitations, the primary one being the relatively low product titer, which is typically seen with transient transfections. In our case, the titers were acceptable for our goals and met very aggressive timelines, but for therapies requiring large clinical doses, the method may not be economical for manufacturing clinical supplies. Cell clumping could also compromise the ability to use transient transfection to its full potential and needs to be carefully evaluated during process development. Overall, one would need to assess economical trade-offs between the time savings afforded by transient transfection and the potential for generating higher-titer product cell lines through stable transfection, which can be very product-specific.

## Figures and Tables

**Table 1:** Comparison of eOD-GT8 60mer nanoparticle product characteristics from research (2.0 L), process development (10 L) and large-scale (200 L) production runs using transient transfection in HEK293H cells.

Method		Research Run (2.0 L)	Process Batch (2 × 10 L)	Non-Clinical Batch (200 L)	cGMP Batch (200 L Scale)
pH		7.2	7.2	7.3	7.2
UV280-content		1.0 mg/mL	1.0 mg/mL	1.1 mg/mL	1.0 mg/mL
ELISA		100%	60%	90%	89%
SE-HPLC		>95%	>92.8%	95.35%	95.81%
DLS		n.d.	n.d.	29.85 nm	26.46 nm
qPCR Residual Host cell DNA		n.d.	n.d.	≤90 ng/mL	≤90 ng/mL
SDS-PAGE (Non-reduced and Reduced)		Four bands ~50 KDa	Four bands ~50 KDa	Four bands ~50 KDa	Four bands ~50 KDa
AUC-Sedimentation Velocity		One main peak (97.8%)	n.d.	One main peak (>95%)	One main peak (>90%)
N-Terminal Edman Sequencing		MQIYEGKLTA *	MQIYEGKLTA *	ETGMQIYEGK ^†^	ETGMQIYEGK ^†^
LC-MS/MS ^‡^	Sequence coverage	97.9%	98%	99.7%	98% ^†^
	Fully occupied N-glycan site	187, 234	187, 234, 267	187, 234, 298	187, 234, 261, 267, 298
	Partially occupied N-glycan site	261, 267, 275, 298, 334	298, 334	261, 267, 282, 315, 334, 339	315, 334, 339
	Fully unoccupied N-glycan site	282, 315, 339	261, 275, 282, 315, 339	275	275, 282
HILIC-FLD-MS/MS		High mannose; 82.9% Man9	High mannose; 90% Man9	High mannose; 95.5% Man9	High mannose; 91.9% Man9
RP-UPLC		Mannose: 89.1% Glucosamine: 10.9%	Mannose: 94.7% Glucosamine: 5.3%	Mannose: 87.3% Glucosamine: 12.7%	Mannose: 92.4% Glucosamine: 7.6%
Endotoxin		n.d.	≤100 EU/mL	≤100 EU/mL	≤100 EU/mL
Bioburden		n.d.	≤1 CFU per 10 mL (TSA) ≤1 CFU per 10 mL (SDA)	≤1 CFU per 10 mL (TSA) ≤1 CFU per 10 mL (SDA)	≤1 CFU per 10 mL (TSA) ≤1 CFU per 10 mL (SDA)

* N-terminal sequence MQIYEGKLTA determined using N-terminal sequencing. ^†^ N-terminal sequence ETGMQIYEGK, determined using N-terminal sequencing, after cleavage of the leader sequence, the ETG residues from the secretion leader sequence remain. ^‡^ Residues are listed in the table in eOD-GT8 60mer consecutive numbering, with MQI as the first three residues. HxB2 numbering corresponds to eOD-GT8 60mer numbering as follows: eOD-GT8 60mer: 187, 234, 261, 267, 275, 282, 298, 315, 334, 339; HxB2: 448, 262, 289, 295, 332, 339, 356, 373, 392, 397. n.d = not done.

## Data Availability

Data are contained within the article.
